# Effects of photobiomodulation therapy in aerobic endurance training and detraining in humans: Protocol for a randomized placebo-controlled trial

**DOI:** 10.1097/MD.0000000000015317

**Published:** 2019-05-03

**Authors:** Paulo Roberto Vicente de Paiva, Heliodora Leão Casalechi, Shaiane Silva Tomazoni, Caroline dos Santos Monteiro Machado, Eduardo Foschini Miranda, Neide Firmo Ribeiro, Amanda Lima Pereira, Amanda Sampaio da Costa, Luana Barbosa Dias, Bianca Cristina Gomes Souza, Matheus Marinho Aguiar Lino, Paulo de Tarso Camillo de Carvalho, Ernesto Cesar Pinto Leal-Junior

**Affiliations:** a Laboratory of Phototherapy and Innovative Technologies in Health; b Post Graduate Program in Rehabilitation Sciences, Nove de Julho University; c Masters and Doctoral Programs in Physical Therapy, Universidade Cidade de São Paulo (UNICID), São Paulo - SP, Brazil.

**Keywords:** aerobic endurance, performance, phototherapy

## Abstract

**Introduction:**

Over the last 10 years, it has been demonstrated that photobiomodulation therapy (PBMT), also known as phototherapy, using low-level laser therapy (LLLT) and/or light-emitting diode therapy (LEDT) has ergogenic effects, improving athletic performance and also accelerating post-exercise recovery. However, many aspects related to these effects and its clinical applicability remain unknown. Therefore, the aim of this project is to evaluate the ergogenic effects of PBMT in detraining after an aerobic endurance training protocol.

**Methods and analyzes:**

A randomized, triple-blind, placebo-controlled clinical trial will be carried out. Healthy male volunteers will be randomly distributed into 4 experimental groups: PBMT before and after training sessions + PBMT during detraining, PBMT before and after training sessions + placebo during detraining, placebo before and after training sessions + PBMT during detraining, and placebo before and after training sessions + placebo during detraining. The aerobic endurance training sessions will be carried out using motorized treadmills during 12 weeks, and the detraining period will consist in the next 4 weeks after that. It will be analyzed the time until exhaustion, maximal oxygen uptake (VO_2max_), and fat percentage of volunteers.

**Discussion:**

Despite the increasing body of evidence for the use of PBMT as an ergogenic agent, several aspects remain unknown. The findings of this study will contribute to the advance of knowledge in this field regarding clinical applications.

**Ethics and dissemination:**

This study was approved by the Research Ethics Committee of Nove de Julho University. The results from this study will be further disseminated through scientific publications in international peer-reviewed journals and presentations at national and international scientific meetings.

**Trial registration number:**

NCT03879226.

## 1 Introduction

Photobiomodulation therapy (PBMT), also known as phototherapy, is a nonthermal process where light interacts with chromophores leading to photo-induced reactions in different tissues. PBMT uses nonionizing light sources, such as lasers, light-emitting diodes (LEDs), and broadband light, from the visible to the infrared spectrum.^[1]^

In 2008, the first randomized controlled trial (RCT) investigating the use of PBMT for athletic performance enhancement was published.^[2]^ This pioneer RCT showed for the very first time that the use of PBMT before an exercise session could enhance athletic performance of high-level athletes, as well as decrease the delayed onset muscle fatigue and prevent the expected increase of blood lactate levels.

Subsequently, a series of clinical studies involving athletes from different sports and healthy individuals conducted by our research group and others showed that, when applied before an exercise session, PBMT can increase the number of repetitions performed,^[2–4]^ lengthen the time to exhaustion,^[2,5–7]^ increase peak torque,^[4,8–11]^ and even improve performance of athletes in field tests,^[12]^ in simulated^[13]^ and real matches.^[14]^ Moreover, studies have also shown that PBMT can potentiate gains in different training protocols, such as strength training^[15,16]^ and aerobic training.^[17]^

Despite the remarkable, and fast progress of the knowledge in this field, which culminated in the publication of 3 systematic reviews^[18–20]^ with meta-analyses^[19,20]^ evidencing the ergogenic effects of PBMT, there are some key aspects that remain unknown and must be investigated. Among them, we highlight the potentially beneficial effects of PBMT during the detraining period after aerobic endurance training protocols.

Various clinical training methods and protocols for performance enhancement, health promotion, and rehabilitation are used by different groups, including athletes, physically active individuals, sedentary individuals, and patients with different disorders and diseases. Among the training types and protocols, aerobic endurance training stands out. However, training protocols very often require discontinuation due to illness, injury, or other factors that may alter the capacity for physical activity, thereby causing a rapid loss of aerobic endurance conditioning,^[21]^ which represents a challenging problem in clinical practice. It should also be noted that, thus far, no evidence or consensus on any particular resource or method that may decrease the effects of detraining has been found.

An interesting study conducted by Wasik et al^[22]^ assessed the effects of PBMT on the oxidative metabolism of peripheral blood cells (erythrocytes, granulocytes, and lymphocytes) by irradiating heparinized blood samples from 15 volunteers. After irradiation, partial pressure of oxygen (PO_2_) and oxygen saturation (SaO_2_) increased, which shows that PBMT-induced photochemical reactions in blood cells may enhance the oxygen-carrying ability of blood. These interesting findings suggest that PBMT may attenuate the loss of performance observed during the detraining period after an aerobic endurance training program because oxygen transport and, consequently, the ability of muscles to use it, decrease during the detraining period.

Therefore, the effects of PBMT on aerobic endurance detraining remain unknown and it is a scientific challenge that will represent a key advance in the knowledge of this field when it is overcome. With this aspect in mind, this project aims to assess the ergogenic effects of PBMT during the detraining period after completing an aerobic endurance training protocol.

## 2 Methods and analyses

### 2.1 Design and ethical aspects

A randomized, triple-blind (volunteers, therapists, and assessors), placebo-controlled clinical trial will be performed at Laboratory of Phototherapy and Innovative Technologies in Health. The study will follow the ethical guidelines and was approved by the Research Ethics Committee of Nove de Julho University (protocol number 1781602). Moreover, this protocol was prospectively registered at ClinicalTrials.org (NCT03879226). All volunteers will sign an informed consent at the time of enrolment in this study.

### 2.2 Subjects and sample size

As no studies assessing the effects of PBMT during the detraining period after an aerobic training program are currently available, the number of participants per group in the present study was calculated based on a pilot study, with 5 volunteers per group, which was conducted by our research group in order to estimate the sample size. A β value of 20% and an α of 5% were used to calculate the sample size.

The pilot study showed that applying PBMT during the detraining period resulted in a time to exhaustion (primary outcome of this study) of 923.60 seconds (65.77 standard deviation) during the progressive treadmill test, whereas applying the placebo during the detraining period resulted in a time to exhaustion of 846.82 seconds (99.23 standard deviation). We used the Researcher's Toolkit to calculate the sample (https://www.dssresearch.com/KnowledgeCenter/toolkitcalculators/samplesizecalculators.aspx).

On the basis of the aforementioned parameters used to calculate the sample, we found 15 volunteers per group, for a total of 60 volunteers. Therefore, predicting a 20% sample loss, 72 healthy, physically inactive male volunteers aged from 18 to 35 years will be recruited for the study to ensure a final sample size of 60 volunteers. As the PBMT device used in the study causes no harmful thermal effects,^[23]^ volunteers of different skin colors will be recruited.

Volunteers will be informed about all study procedures and will be asked to sign the Informed Consent Form before their enrollment in the study.

### 2.3 Eligibility criteria

Healthy men aged from 18 to 35 years with no history of musculoskeletal injury in the hip and knee regions in the 2 months before the study who do not regularly use pharmacological agents and/or nutritional supplements and who complete at least 80% study procedures will be included in the study. Volunteers who show any musculoskeletal injury in the 2 months before the study or who become injured during the study, who regularly use any type of nutritional supplement of pharmacological agent, or who show signs and symptoms of any neurological, metabolic, inflammatory, pulmonary, oncological, or cardiovascular disease that may limit the execution of high-intensity exercises will be excluded from the study.

### 2.4 Randomization and blinding and experimental groups

To avoid selection bias and to ensure that all individuals are randomly allocated to any group, balanced block randomization will be performed based on the primary outcome (time to exhaustion in the progressive treadmill test) by a researcher who will have no contact with the study subjects or the other researchers involved in the project.

A researcher will program the device (PMBT or placebo) and will be instructed not to inform the volunteers or other researchers as to the type of treatment (PMBT or placebo). Therefore, the researcher responsible for the treatment will be blinded to the type of treatment being administered to the volunteers. The sounds and signals emitted from the device as well as the information displayed on the screen will be identical, regardless of the type of treatment (PMBT or placebo).

Randomization labels will be created through the random.org website, and a series of sealed, opaque, and numbered envelopes will be used to ensure confidentiality and will determine to which experimental group each volunteer will be allocated. Volunteers will be allocated as described below:

**A**: PBMT before and after the aerobic training sessions (12 weeks, 3 times a week) and PBMT during the detraining period (4 weeks, 3 times a week).
**B:** PBMT before and after the aerobic training sessions (12 weeks, 3 times a week) and placebo during the detraining period (4 weeks, 3 times a week).
**C:** Placebo before and after the aerobic training sessions (12 weeks, 3 times a week) and PBMT during the detraining period (4 weeks, 3 times a week).
**D:** Placebo before and after the aerobic training sessions (12 weeks, 3 times a week) and placebo during the detraining period (4 weeks, 3 times a week).

The individuals randomly allocated to the different groups will be subjected to 12 consecutive weeks of aerobic endurance training in a motorized treadmill, with 3 training sessions per week on nonconsecutive days.

After the 12-week training period, the volunteers will receive the treatment, either PBMT or placebo, depending on the group to which they were allocated, during 4 weeks (3 times a week) without training.

**Figure 1:**
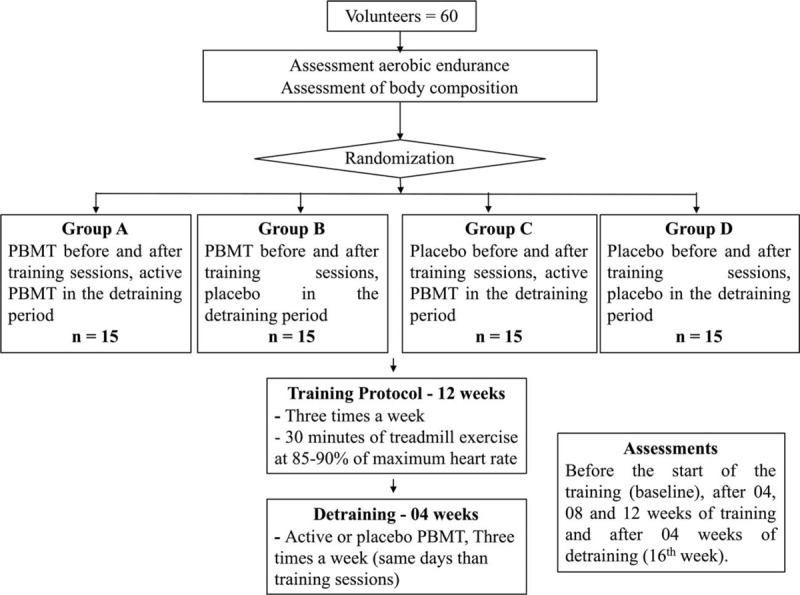
CONSORT flowchart.

The evaluations described below will be performed before starting the protocol (baseline) and after 4, 8, and 12 weeks of aerobic endurance training, as well as after 4 weeks without training (detraining period) at 16th week. A flowchart summarizing the procedures of this study is presented in Fig. 1.

### 2.5 Procedures

#### 2.2.1 Cardiorespiratory evaluation

Ergospirometry is currently the most accurate cardiorespiratory fitness test.^[24]^ In this study, we will use a progressive treadmill protocol, previously used by our research group,^[5,7,17]^ an ergometric treadmill model Super ATL, and a gas analyzer model VO2000 (Inbrasport, Porto Alegre - RS, Brazil), which will be connected to a microcomputer for data visualization and recording. The ergospirometry test will start with the treadmill set at 1% slope, with an initial speed of 3 km/h, which will be maintained until the end of the warm-up phase lasting 3 minutes. After the warm-up phase, the speed will be increased by 1 km/h every minute until reaching a maximum speed of 16 km/h. The end of the test will be defined by the volunteers, who will be instructed to perform the test until they reach exhaustion.^[5,7,17]^ Then, the recovery phase will begin, lasting 3 minutes at a speed of 6 km/h. In the test, data on the total time of the exercise (time to exhaustion), maximum oxygen uptake in relative values in relation to body mass (VO_2max_), and aerobic and anaerobic thresholds will be recorded.^[5,7,17]^ These data will be used to assess the performance of the subjects in the exercise protocol, as they are currently the parameters most used for this purpose in the literature.^[24]^

The entire test will be monitored by electrocardiography and blood pressure measurements. If any abnormal changes in heart rate or in blood pressure are found or if the volunteer has any complaint, the test will be discontinued and the volunteer will be excluded from the study.

#### 2.2.2 Body composition evaluation

All body composition evaluations will be performed by the same technician (level II of the International Society for the Advancement of Kineanthropometry – ISAK), using the procedures established by the ISAK.^[25]^ Height, body mass, lengths of body segments, diameters, perimeters, and skinfolds will be measured to assess muscle mass, adipose mass, residual mass, bone mass, and epithelial mass.^[17]^

#### 2.2.3 Aerobic training

Aerobic training will be performed on a treadmill, with and without applying PBMT, 3 times a week on nonconsecutive days, for 12 weeks. Each training session will last 30 minutes, with an intensity ranging from 85% to 90% maximum heart rate,^[17]^ which will be assessed using the cardiorespiratory evaluation protocol described above.^[7,17]^ This protocol will be interrupted when criteria established by the American Heart Association guidelines are met. The subjects will also be evaluated using the 0 to 10 Borg scale, which is a simple method for classifying perceived exertion, feeling of physical fatigue, or dyspnea.

#### 2.2.4 Photobiomodulation therapy (PBMT)

**Figure 2:**
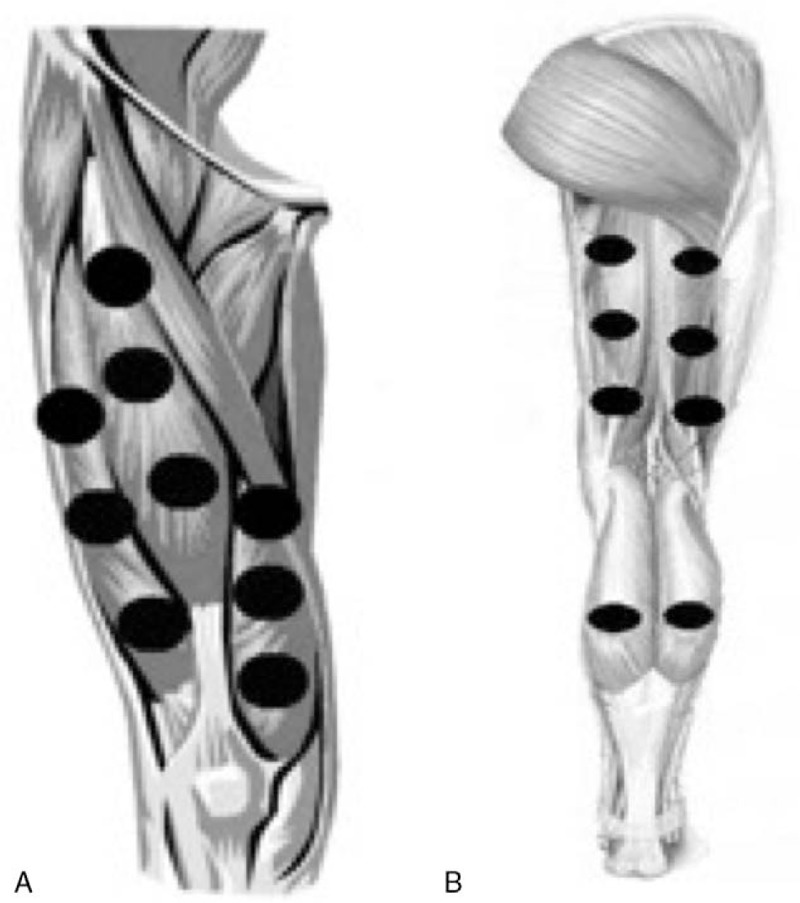
(A) Irradiation sites for PBMT in anterior thigh muscles. (B) Irradiation sites for PBMT in posterior thigh muscles and plantar flexor muscles.

PBMT or placebo will be applied before and after each training session. This irradiation protocol was tested and optimized in a previous study conducted by our research group.^[17]^ The results of this study^[17]^ showed that PBMT before and after each training session was the most effective in enhancing the effects of aerobic training. PBMT will be applied bilaterally using the direct contact method with light pressure on the skin at different sites, namely 9 sites on the knee extensor muscles (Fig. 2A), 6 sites on the knee flexor muscles, and 2 sites on the plantar flexor muscles (Fig. 2B).

To apply PBMT, a 12-diode cluster will be used, including four 905 nm laser diodes (12.5 W peak power of each diode, 250 Hz), four 875 nm LED diodes (17.5 mW average power of each diode), and four 640 nm LED diodes (15 mW average power of each diode), manufactured by Multi Radiance Medical (Solon, OH). Considering the large irradiation area used in the present project, the use of diode clusters will be essential for the application of PBMT. In this study, the cluster is circular and has a total irradiation area of 20 cm^2^.

The dose used for active PBMT will be 30 J per area (228 seconds of irradiation in each area),^[10]^ 510 J irradiated energy per lower limb,^[7,12,17]^ and 1020 J total irradiated energy.^[7,12,17]^ The dose that will be used at each site was previously tested and optimized by our research group using the same PBMT device with favorable results in terms of enhancing performance and muscle recovery.^[10]^ Furthermore, the irradiation sites were also previously optimized by our research group.^[7,12,17]^ The complete description of PBMT parameters is presented in Table 1.

**Table 1:**
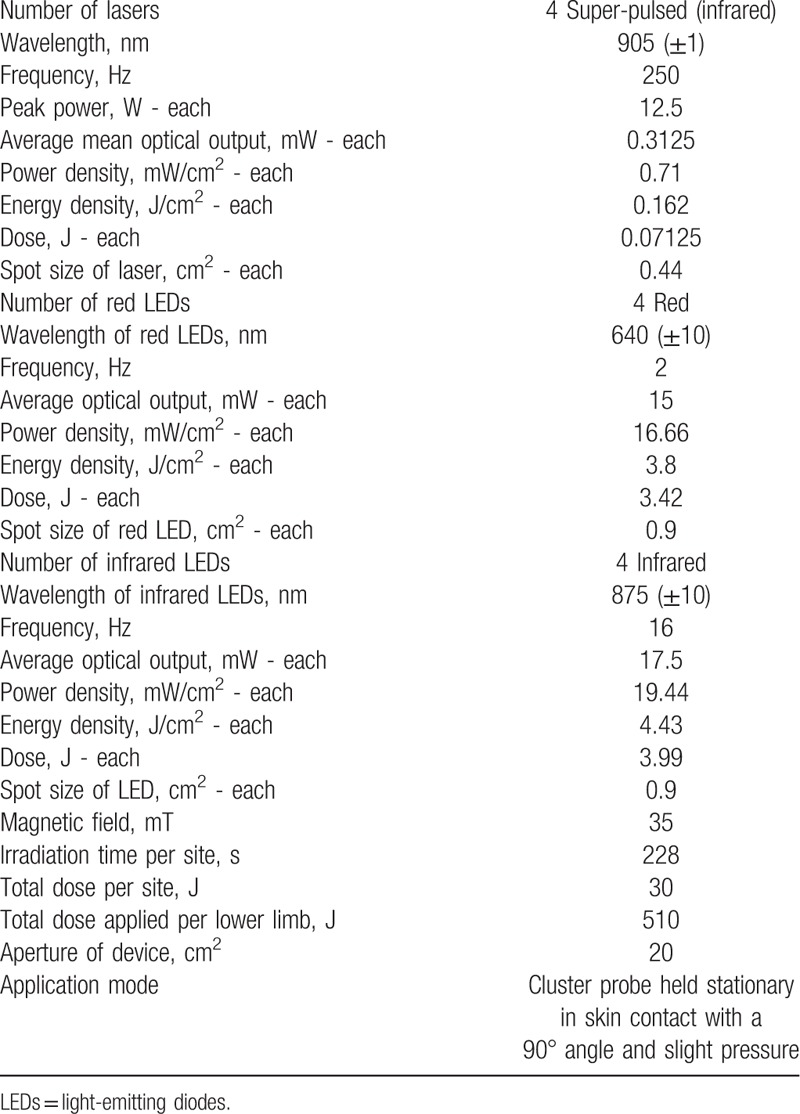
Parameters for PBMT.

### 2.6 Statistical analysis

The primary outcome will be time until exhaustion obtained from progressive treadmill test. The secondary outcomes are VO_2max_ and fat percentage test. The intention-to-treat analysis will be followed a priori. All the data will be analyzed by a blinder researcher not involved in data collection. The findings will be tested for their normality using the Shapiro–Wilk test. Parametric data will be expressed as mean and standard deviation and nonparametric data as median and respective upper and lower limits. Parametric data will be analyzed by 2-way repeated measures analysis of variance (ANOVA; time vs experimental group) with post-hoc Bonferroni correction. Nonparametric data will be analyzed using the Friedman test and, secondarily, the Wilcoxon signed-rank test. Data will be analyzed in terms of both their absolute values and their percentage of change based on the values established in the baseline. The significance level will be set at *P* < .05.

### 2.7 Data availability

The datasets generated and analyzed during the current study will be available from the corresponding author upon reasonable request.

## 3 Discussion

The effects of PBMT on the maintenance of gains from an aerobic endurance training protocol during a period of discontinuation of physical activity, that is, a detraining period, have not yet been investigated. We believe that PBMT will have a positive effect on the maintenance of gains from aerobic endurance training, during the detraining period. Given that that many individuals, whether they are athletes or not, often fail to maintain training routines, the ability to minimize this loss would be of significant value in terms of accelerating the return to their previous fitness levels.

As strengths of this study, we can highlight that it has high methodological quality, as it is a randomized, controlled, and prospectively registered clinical trial. Another strength is that it has a triple blinded design, which means that outcome assessors, therapists, and patients will be blinded to interventions over the course of the study.

In addition, the sample size was calculated based on a pilot study to provide the appropriate statistical power to detect precise differences for the primary outcome of the study. And finally, the doses, sites of application, and the moment to apply PBMT were previously optimized and published in international peer-reviewed journals.

Despite this study will analyze the effects of PBMT only until 4 weeks of detraining after 12 weeks of strength training, it is important to stress that such aspect never was investigated previously. And therefore, we believe that the present project is a major step toward the widespread use of PBMT as an ergogenic agent for not only increasing performance but also decreasing losses incurred by detraining. These factors have a direct impact on clinical practice, not only to athletes but also to patients in physical rehabilitation programs, when performing aerobic endurance training is not possible.

## 4 Dissemination policy

The results from this study will be disseminated through scientific publications in international peer-reviewed journals and presentations at national and international scientific meetings.

## Author contributions

PRVP, HLC, and ECPL-J contributed to the concept and design of the study and established the hypothesis and wrote the original proposal. PRVP, HLC, SST, CSMM, EFM, NFR, ALP, ASC, LBD, BCGS, MMAL, PTCC, and ECPL-J contributed significantly in creating the manuscript. SST and PTCC performed critical revisions of the manuscript. ECPL-J wrote the final version of the manuscript. All authors read and approved the final version of the manuscript.

**Conceptualization:** Paulo Roberto Vicente de Paiva, Shaiane Silva Tomazoni, Paulo de Tarso Camillo de Carvalho, Ernesto Cesar Pinto Leal-Junior.

**Data curation:** Caroline dos Santos Monteiro Machado.

**Formal analysis:** Caroline dos Santos Monteiro Machado, Eduardo Foschini Miranda.

**Funding acquisition:** Paulo Roberto Vicente de Paiva, Ernesto Cesar Pinto Leal-Junior.

**Investigation:** Heliodora Leão Casalechi, Neide Firmo Ribeiro, Amanda Lima Pereira, Amanda Sampaio da Costa, Luana Barbosa Dias, Bianca Cristina Gomes Souza, Matheus Marinho Aguiar Lino.

**Methodology:** Paulo de Tarso Camillo de Carvalho.

**Project administration:** Heliodora Leão Casalechi, Caroline dos Santos Monteiro Machado, Ernesto Cesar Pinto Leal-Junior.

**Supervision:** Ernesto Cesar Pinto Leal-Junior.

**Writing – original draft:** Paulo Roberto Vicente de Paiva, Heliodora Leão Casalechi, Shaiane Silva Tomazoni, Caroline dos Santos Monteiro Machado, Eduardo Foschini Miranda, Neide Firmo Ribeiro, Amanda Lima Pereira, Amanda Sampaio da Costa, Luana Barbosa Dias, Bianca Cristina Gomes Souza, Matheus Marinho Aguiar Lino, Paulo de Tarso Camillo de Carvalho, Ernesto Cesar Pinto Leal-Junior.

**Writing – review & editing:** Shaiane Silva Tomazoni, Ernesto Cesar Pinto Leal-Junior.

Ernesto Cesar Pinto Leal-Junior orcid: 0000-0001-6393-7616.
